# Is Habitat More Important than Phylogenetic Relatedness for Elucidating the Gut Bacterial Composition in Sister Lizard Species?

**DOI:** 10.1264/jsme2.ME21087

**Published:** 2022-06-30

**Authors:** Mauricio Hernández, Sergio Ancona, Aníbal H. Díaz De La Vega-Pérez, Ligia C. Muñoz-Arenas, Stephanie E. Hereira-Pacheco, Yendi E. Navarro-Noya

**Affiliations:** 1 Doctorado en Ciencias Biológicas, Centro Tlaxcala de Biología de la Conducta, Universidad Autónoma de Tlaxcala, Carretera Tlaxcala-Puebla km 1.5, 90062, Tlaxcala, Mexico; 2 Departamento de Ecología Evolutiva, Instituto de Ecología, Universidad Nacional Autónoma de México, C.P., 04510, Mexico City, Mexico; 3 Consejo Nacional de Ciencia y Tecnología-Centro Tlaxcala de Biología de la Conducta, Universidad Autónoma de Tlaxcala, Carretera Tlaxcala-Puebla km 1.5, 90062, Tlaxcala, Mexico; 4 Facultad de Ingeniería Ambiental, UPAEP, Puebla, Mexico; 5 Estación Científica La Malinche, Centro Tlaxcala de Biología de la Conducta, Universidad Autónoma de Tlaxcala, Carretera Tlaxcala-Puebla km 1.5, 90062, Tlaxcala, Mexico; 6 Laboratorio de Interacciones Bióticas, Centro de Investigación en Ciencias Biológicas, Universidad Autónoma de Tlaxcala, Carretera San Martín Texmelucan km 10.5, 90120, San Felipe Ixtacuixtla, Tlaxcala, Mexico

**Keywords:** gut microbiota, mountain ecosystem, related species, reptile microbiome, wild lizard

## Abstract

The gut microbiota influences the phenotype and fitness of a host; however, limited information is currently available on the diversity and functions of the gut microbiota in wild animals. Therefore, we herein examined the diversity, composition, and potential functions of the gut microbiota in three *Sceloporus* lizards: *Sceloporus aeneus*, *S. bicanthalis*, and *S. grammicus*, inhabiting different habitats in a mountainous ecosystem. The gut bacterial community of *S. bicanthalis* from alpine grasslands at 4,150‍ ‍m a.s.l. exhibited greater taxonomic, phylogenetic, and functional alpha diversities than its sister species *S. aeneus* from cornfields and human-induced grasslands at 2,600‍ ‍m‍ ‍a.s.l. Bacteria of the genus *Blautia* and metabolic functions related to the degradation of aromatic compounds were more abundant in *S. bicanthalis* than in *S. aeneus*, whereas *Oscillibacter* and predicted functions related to amino acid metabolism and fermentation were more abundant in *S. aeneus*. The structure of the dominant and most prevalent bacteria, i.e., the core microbiota, was similar between the sister species from different habitats, but differed between *S. grammicus* and *S. aeneus* cohabiting at 2,600‍ ‍m‍ ‍a.s.l. and between *S. grammicus* and *S. bicanthalis* cohabiting at 4,150‍ ‍m a.s.l. These results suggest that phylogenetic relatedness defines the core microbiota, while the transient, *i.e.*, non-core, microbiota is influenced by environmental differences in the habitats. Our comparisons between phylogenetically close species provide further evidence for the specialized and complex associations between hosts and the gut microbiota as well as insights into the roles of phylogeny and ecological factors as drivers of the gut microbiota in wild vertebrates.

The gut microbiota strongly influences the health of its vertebrate hosts via energy and nutrient acquisition (Matsuyama *et al.*, 2019) or protection against pathogens, either by competing against pathogenic microbes or by boosting the host’s immune system (Belkaid and Hand, 2014). Importantly, the gut microbiota shapes the phenotype of the host, and, thus, plays a critical role in how natural populations respond to environmental conditions (Alberdi *et al.*, 2016). Nevertheless, the majority of research on the composition and functions of the vertebrate gut microbiota have focused on mammals, and predominantly on humans and captive mammalian populations (Colston and Jackson, 2016). Therefore, limited information is currently available on the composition and functions of the gut microbiota in wild vertebrate populations.

Non-avian reptiles are taxonomically very diverse (Uetz and Hošek, 2021), and are widely distributed and play important ecological functions in their habitats (Pereira de Miranda, 2017); however, research on the composition and functions of their gut microbial communities is in its infancy. Only a few studies have examined the wild and captive reptilian gut microbiota, and the findings obtained showed that several factors may influence gut microbiota variations in this group of animals, *e.g.*, climate change (Bestion *et al.*, 2017), an altitudinal gradient (Zhang *et al.*, 2018; Montoya-Ciriaco *et al.*, 2020), gestation (Trevelline *et al.*, 2019), diet and captivity (Kohl *et al.*, 2017), multiple mating (White *et al.*, 2011), and phylogeny and ecomorphism (Ren *et al.*, 2016).

Coevolution between gut microbial communities and hosts has been documented in mammals (Li *et al.*, 2017; Ingala *et al.*, 2018), reptiles (Scheelings *et al.*, 2020), and birds (Sottas *et al.*, 2021), with the general pattern being that the gut microbiota is more similar in closely related species than among distantly related species. Nevertheless, similarities in the composition of the gut microbiota of phylogenetically close species may not be unambiguously dissociated from ecological similarities between hosts. A previous study reported that the gut bacterial composition did not significantly differ between sympatric populations of closely related species of the deer mice *Peromyscus leucopus* and *P. maniculatus gracilis*, which have similar diets (Baxter *et al.*, 2015). These findings raised the question as to whether this was due to ecological similarities rather than phylogenetic relatedness between host species (see Sottas *et al.*, 2021 for a similar example in the nightingale birds *Luscinia megarhynchos* and *L. luscinia*).

Reptiles provide striking examples of the complex relationships between the composition of the gut microbiota and the ecology and phylogeny of hosts. Small dietary variations may explain differences in the gut microbiota compositions and structures of two *Liolaemus* lizard species (*Liolaemus parvus* and *L. ruibali*; Kohl *et al.*, 2017). Similarly, fine-scale exposure to different local pools of microbial species resulted in differences in the gut microbial communities of the land iguanas *Conolophus subcristatus* and *C. pallidus* cohabiting the Galápagos islands (Hong *et al.*, 2011; Lankau *et al.*, 2012). Variations in gut bacterial communities were detected between two species of anoles, *Anolis cristatellus* and *A. sagrei*, which exhibit convergent trunk-ground ecomorphs (Ren *et al.*, 2016). However, further research on other reptiles is needed to confirm the generality of these patterns.

In the present study, we used 16S rRNA gene sequencing to compare the taxonomic, phylogenetic, and functional diversities of the fecal bacterial biota (hereafter referred to as the gut microbiota) of two closely related lizard species of the genus *Sceloporus* (*Phrynosomatidae*): the oviparous lizard *Sceloporus aeneus* Wiegmann, 1828, and the viviparous lizard *S. bicanthalis* Smith, 1937 inhabiting the volcano La Malinche (4,460‍ ‍m a.s.l.) in the Trans-Mexican Volcanic Belt. These sister species diverged from their common ancestor ~5.5 million years ago (Wiens *et al.*, 2013), and exhibit similar morphologies and body sizes (snout to vent length 51–59‍ ‍mm). Both species are terrestrial and inhabit grasslands (Méndez de la Cruz *et al.*, 2018), their maximum average lifespan is approximately one year (Rodríguez-Moreno, 2004), and they are generalist insectivorous (Canseco-Márquez and Gutiérrez-Mayén, 2010; Cruz-Elizalde *et al.*, 2021). In La Malinche, these lizard species occupy contrasting habitats. *S. aeneus* is mainly found in cornfields, human-induced grasslands, and shrubs located at 2,600‍ ‍m a.s.l., with a mean air temperature of 13.20±6.69°C and mean relative humidity of 66.68±22.09% (Domínguez-Godoy *et al.*, 2020). In contrast, *S. bicanthalis* is mainly found in alpine grasslands located at 4,150‍ ‍m a.s.l., where mean air temperature is 6.02±4.7°C and mean relative humidity is 67.74±29.93% (Domínguez-Godoy *et al.*, 2020).

After the gut microbiota of *S. aeneus* was shown to differ from that of *S. bicanthalis* despite them being sister species, we compared the core gut microbiota of these species with that of another member of the genus *Sceloporus*, the mesquite lizard *S. grammicus* Wiegmann, 1828, which coexists with both species in the studied sites. *S. grammicus* is an insectivorous lizard with arboreal and saxicolous habits that lives at 2,300–4,400‍ ‍m a.s.l. in La Malinche (Díaz de la Vega-Pérez *et al.*, 2019a). We speculated that if the core gut bacterial composition differs between *S. grammicus* and the two other *Sceloporus* species, then differences in the gut bacterial composition between *S. aeneus* and *S. bicanthalis* may be attributed to species identity (Sottas *et al.*, 2021) rather than differences in the ecological conditions to which these lizards are subject. Data on the core gut microbiota of *S. grammicus* were taken from Montoya-Ciriaco *et al.* (2020).

## Materials and Methods

### Ethics statement

The sampling and handling of lizards complied with ethical and legal regulations in Mexico to conduct research on wild organisms, as stipulated in the Norma Oficial Mexicana (NOM-126-ECOL-2000). Permission for the sampling and handling of lizards was granted by the Secretaría de Medio Ambiente y Recursos Naturales (SEMARNAT, Mexico) under the collecting permits SGPA/DGVS/15396/15 and SGPA/DGVS/007736/20.

### Study area and sampling

La Malinche is an eroded stratovolcano situated in the Mexican states of Tlaxcala and Puebla (N 19°, 14′ W 98°02′). This volcano is mainly covered by cornfields, shrubs, and herbaceous plants (low-zone at 2,600‍ ‍m a.s.l.), coniferous (*Pinus* spp. and *Abies* spp.) and oak (*Quercus* spp.) forests (medium-zone at 3,200‍ ‍m a.s.l.), and rocky alpine grassland and shrubs of *Juniperus monticola* (high-zone at 4,150‍ ‍m a.s.l.) (Domínguez-Godoy *et al.*, 2020). Lizards were sampled in February 2020 at different elevations: 9 individuals of *S. aeneus* were collected at 2,600‍ ‍m a.s.l. (19°12′‍ ‍N, 97°55′ W) and 9 of *S. bicanthalis* at 4,150‍ ‍m a.s.l. (19°14′ N, 98°01′ W). Lizards were captured by hand between 0900 and 1600‍ ‍h. Each captured specimen was transported individually to the La Malinche Scientific Station, located at 3,100‍ ‍m a.s.l. (19°14′‍ ‍N, 97°59′ W), for fecal sampling. Lizards were housed individually in sanitized cages and maintained at 20–25°C until they naturally defecated. The base of each cage was covered with a sterile paper sheet and fecal samples were collected with sterile forceps. Fecal samples were placed in separate 1.5-mL sterile polypropylene tubes, stored and transported into a cooler with ice (<4°C), and then kept at –20°C until DNA extraction. All lizards were released alive in good physical condition at the site at which they were captured.

### DNA isolation and library preparation

DNA was extracted from feces using two different methods of cell lysis and pooled as previously described by Montoya-Ciriaco *et al.* (2020). DNA quality was verified by electrophoresis through 1% agarose gels. Amplification of the V3–V4 region of the 16S rRNA gene was performed using the 341F (5′-CCTACGGGNGGCWGCAG-3′) and 805R (5′-ACHVGGGTATCTAATCC-3′) primers (Herlemann *et al.*, 2011) modified with adapters for the Illumina sequencing platform. The thermal cycling conditions of PCR were as follows: denaturation at 95°C for 2‍ ‍min, followed by 28 cycles of denaturation at 95°C for 30‍ ‍s, annealing at 55°C for 30‍ ‍s, elongation at 72°C for 30‍ ‍s, and a final extension at 72°C for 5‍ ‍min. A negative control was included in each PCR to detect reagent contamination. PCR was performed in triplicate, pooled, purified using the FastGene Gel/PCR Extraction Kit (Nippon Genetics), quantified using a NanoDrop 3300 fluorospectrometer (Thermo Fisher Scientific) with the PicoGreen dsDNA assay (Invitrogen), and combined at equal molar concentrations. Sequencing was conducted by Macrogen with 300-bp PE MiSeq runs (Illumina). Raw sequence databases are available at the Sequence Read Archive (SRA) from the NCBI under the project number PRJNA816478.

### Bioinformatic ana­lysis

A sequencing data ana­lysis was performed using the open-source software QIIME. Demultiplexing was conducted with QIIME v1.9.1 (Caporaso *et al.*, 2010). Sequences were imported into QIIME2 v2021.4.0 (Bolyen *et al.*, 2019). Denoising, quality filtering, trimming, paired-end sequence merging, dereplication in Amplicon Sequence Variants (ASVs), and chimera filtering were performed with the DADA2 plugin (Callahan *et al.*, 2016). Standard filtering parameters (maxEE=2, truncQ=2, p-pooling-method=pseudo) were applied to forward and reverse reads, and forward reads were trimmed to 260 nt and reverse to 200 nt. Query sequences (rep-set) were assigned taxonomically with classify-sklearn with a Naive Bayes supervised learning algorithm using the trained SILVA 16S rRNA gene database version v.138.1. Organellar 16S rRNA sequences, *i.e.*, from mitochondria and chloroplasts, were eliminated. After these filtering steps, samples with fewer than 1,000 reads were eliminated from the dataset. To construct the phylogeny for the calculation of phylogenetic diversity, the rep-set was aligned with MAFFT (Katoh and Standley, 2013) and a rooted maximum likelihood tree was built using IQ-TREE multicore version 2.0.3 (Minh *et al.*, 2019) with the best substitution model for our dataset as selected with the ModelFinder algorithm, *i.e.*, the GTR+F+R10 model. The potential functions of the microbiome were investigated with PICRUSt2. The rep_set and a reference database of genomes from the Integrated Microbial Genomes database were aligned with hidden Markov models to insert ASVs into a reference tree. Genome predictions were performed with a hidden-state algorithm. Pathway abundance based on Enzyme Classification number (EC number) abundance was inferred with MetaCyc (Caspi *et al.*, 2016).

### Statistical ana­lysis

Downstream statistical ana­lyses were performed within the R environment (R Core Team, 2020). We used Hill numbers to measure true taxonomic, phylogenetic, and functional alpha diversities at different *q* diversity orders: *q*=0 corresponds to the total number of ASVs or species richness, *q*=1 corresponds to frequent ASVs and is equivalent to the exponential of the Shannon entropy, and *q*=2 corresponds to dominant ASVs and is equivalent to the reciprocal of the Simpson index (Chao *et al.*, 2014; Alberdi and Gilbert, 2019). Taxonomic, functional, and phylogenetic alpha diversities were obtained using the *hillR* R package (Ma and Li, 2018). Taxonomic alpha diversity was calculated with the frequency table of ASVs. Functional diversity was assessed as the mean functional diversity per species (MD_q), which calculates the effective sum of pairwise distances between a fixed species and all other species using the frequency table of ASVs (community) and that of EC numbers (functional traits). Hill numbers for phylogenetic diversity incorporate the tree’s branching pattern, the relative branch lengths, and the relative abundance of each node/branch, and the unit of measurement is the effective total branch length (Chao *et al.*, 2010). A non-parametric Mann-Whitney-Wilcoxon test was implemented to detect significant differences in alpha diversities between *S. aeneus* and *S. bicanthalis*. Adjusted *P*-values were considered to be significant at *P*<0.05.

Due to the compositional nature of the microbiome data, we applied a centered-log-ratio transformation “*clr*” to the frequency table of ASVs, which makes the data symmetric and linearly related, with the *ALDEx2* R package (Gloor *et al.*, 2017). A Robust Aitchison Principal Component Analysis (RPCA), which is a proper distance metric for compositional data (Aitchison *et al.*, 2000), was obtained to examine variations in bacterial community assemblages, and these differences were assessed using a permutational multivariate ana­lysis of variance (perMANOVA) with 999 permutations using the *vegan* R package (Oksanen *et al.*, 2017). An ANOVA-like Differential Expression (ALDEx) ana­lysis was used to examine differences in the abundance of taxonomic groups and EC numbers among *S. aeneus* and *S. bicanthalis* with the *ALDEx2* R package. Raw counts were used as an input and Monte Carlo Dirichlet instances of *clr* transformation values were generated with the function “*aldex.clr*”. To test for differences in abundance between bacterial taxa, a Mann-Whitney-Wilcoxon test was conducted using the function “*aldex.ttest*”. A Benjamini-Hochberg sequential correction was applied to the resulting *P*-value. Heatmaps of differentially abundant taxa and functions were constructed with the *ComplexHeatmap* R package (Gu *et al.*, 2016).

We compared the gut bacterial community structure of the two populations of *S. grammicus* sampled by Montoya-Ciriaco *et al.* (2020) with *S. aeneus* and *S. bicanthalis*: one population coexisting with *S. aeneus* at 2,600‍ ‍m a.s.l., and another population coexisting with *S. bicanthalis* at 4,150‍ ‍m a.s.l. Sequences from the gut bacterial communities of both populations of *S. grammicus* were obtained from the NCBI (https://www.ncbi.nlm.nih.gov/search/all/?term=PRJNA544140). To reduce the bias of comparing two different datasets, the two fasta files of the representative sequences were clustered with a closed-reference clustering method at a similarity threshold of 97% using VSEARCH within QIIME2 and against the Greengenes 16S rRNA gene database version 13_8 (http://greengenes.lbl.gov/Download/). The resulting OTUs were taxonomically assigned with classify-sklearn and frequency tables of taxonomic compositions at the genus level were used for further ana­lyses. It was necessary to use the core gut bacterial communities instead of the whole bacterial communities of the gut, which include both the core microbiota and non-core microbiota, because samples were collected in different years (*S. grammicus* was sampled in 2015; *S. aeneus* and* S. bicanthalis* were sampled in 2020). The core gut microbiota is more stable over time than the non-core gut microbiota (Huse *et al.*, 2012) and, thus, comparisons of the core microbiota allowed us to reduce the potential confounding effect of interannual variations in the composition of gut bacterial communities. Core gut bacterial communities were defined as bacterial genera with a prevalence >55% in the samples of each species. The resulting frequency table that contained the samples from *S. aeneus*,* S. bicanthalis*, and *S. grammicus* at the genus level was *clr* transformed, and perMANOVA and PCA were performed as described above for the comparisons of interest. A Venn diagram was constructed with the *VennDiagram* R package (Chen and Boutros, 2011). A network was built to show the co-occurrence patterns of the core bacterial genus between the three lizard species using the *NetCoMi* R package (Peschel *et al.*, 2021). Zeros from the observation matrix were replaced with pseudocounts with a predefined value of 0.5 and data was *clr* transformed. Correlations (edges) between nodes (core bacterial genera) were obtained with the *SparCC* function (≥0.3) (Friedman and Alm, 2012). The adjacency matrix was obtained with the function “*graph_from_adjacency_matrix*” from the *igraph* R package (Csardi and Nepusz, 2006). Clusters, components, and hubs were identified based on a fast greedy modularity optimization algorithm. Components with unconnected nodes were removed from the network for visualization. The R scripts for the statistical ana­lysis may be found at GitHub (https://github.com/Steph0522/Sceloporus_species).

## Results

Eighteen fecal samples were used to characterize the gut bacterial communities of *S. aeneus* (*n*=9) and *S. bicanthalis* (*n*=9), and resulted in 107,919 good quality sequences (min frequency=1300, max frequency=14503; Table S1). Sixty-one fecal samples from *S. grammicus* (*n*=38 collected at 2,600‍ ‍m a.s.l. and *n*=23 collected at 4,150‍ ‍m a.s.l.) were used to compare the core gut bacterial communities between *S. grammicus* and *S. aeneus* and between *S. grammicus* and *S. bicanthalis*.

### Alpha diversity of gut bacterial communities

**Fig. 1.**
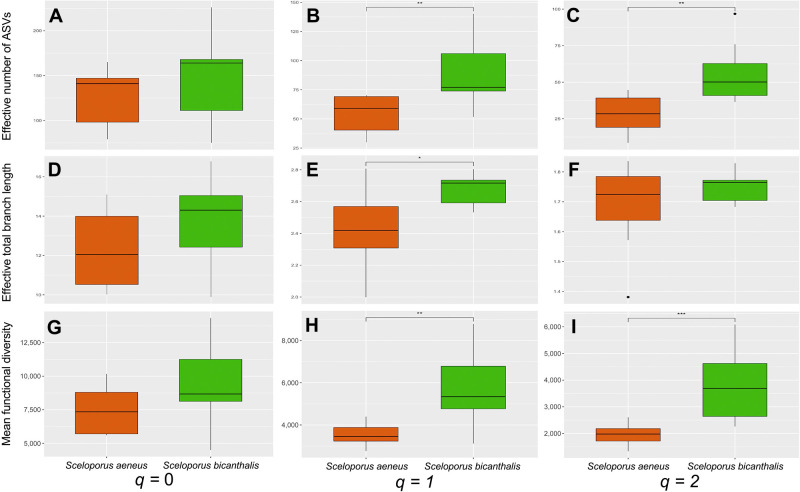
Box and whisker plots (medians, interquartiles, 10–90% percentiles) of the true alpha diversity estimated as Hill numbers of gut bacterial communities of two *Sceloporus* lizard species inhabiting a high-mountain ecosystem. Taxonomic alpha diversity (A, B, C), phylogenetic alpha diversity (D, E, F), and functional alpha diversity (G, H, I) were calculated at diversity orders *q*=0 (A, D, G), *q*=1 (B, E, H), and *q*=2 (C, F, I). Significant differences among lizard species were tested by the Mann-Whitney-Wilcoxon test.

Across all gut bacterial communities, 886 ASVs were identified with an average of 136 ASVs per sample. Except for phylogenetic diversity at *q*=2, the taxonomic, phylogenetic, and functional diversities of gut bacterial communities at *q*=1 and 2 were higher in *S. bicanthalis* than in *S. aeneus* (*P*<0.05; Fig. 1B–I and Table S2). Taxonomic, phylogenetic, and functional richness were similar in both species.

### Taxonomic compositions and structures of gut bacterial communities

**Fig. 2.**
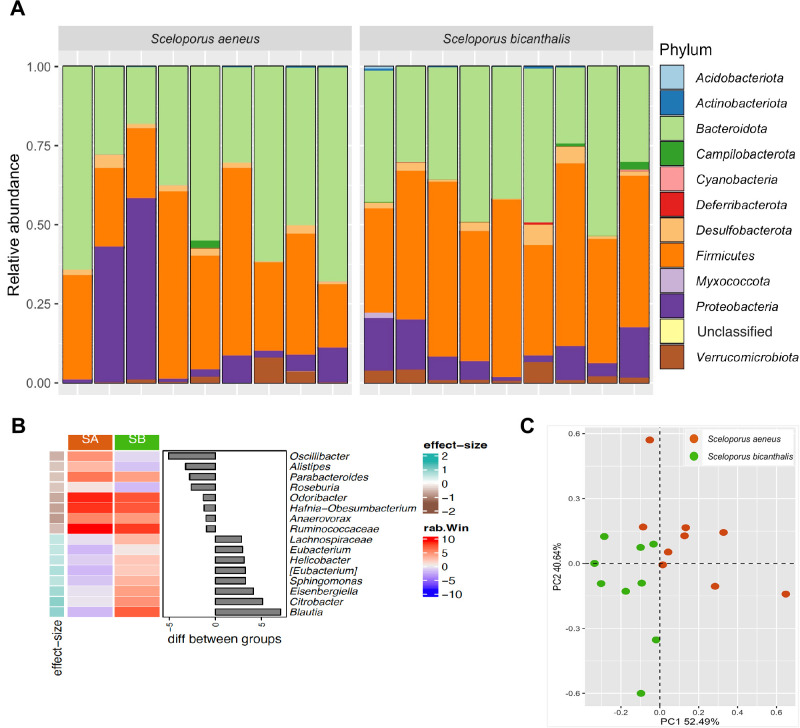
Taxonomic compositions of gut bacterial communities of two *Sceloporus* lizard species inhabiting a high-mountain ecosystem. (A) Bar plot of individual relative abundance at the phylum level, (B) heatmaps with comparisons between *Sceloporus aeneus* (SA) and *Sceloporus bicanthalis* (SB) of the median *clr* value of the 15 most abundant genera, as assessed by an ANOVA-like differential expression tool for compositional data. Bar plots represent the median difference between species, and (C) comparisons of the gut bacterial communities of the *Sceloporus* species from this study by a Robust Principal Component ana­lysis (RPCA).

The gut bacterial communities of *S. aeneus* and *S. bicanthalis* contained 11 bacterial phyla with *Bacteroidota* (42.55±14.50%) being the most abundant, followed by *Firmicutes* (40.71±13.04%), *Proteobacteria* (11.75±15.09%), *Desulfobacterota* (2.16±1.67%), and *Verrucomicrobiota* (2.06±2.32%). The remaining six bacterial phyla had a relative abundance <1% (Fig. 2A). The most abundant genera across all samples were, in decreasing order, *Bacteroides* (19.18±8.48), *Odoribacter* (11.81±5.23), *Parabacteroides* (8.67±6.04), *Hafnia-Obesumbacterium* (7.45±16.47), *Alistipes* (6.77±5.81), [Eubacterium] (3.47±2.70), *Roseburia* (2.95±6.72), and *Akkermansia* (2.62±2.87) (Supplementary Fig. S1). The most abundant genera were also the most prevalent. The core gut bacterial microbiota of *Sceloporus* spp. comprised *Bacteroides*, *Parabacteroides*, *Odoribacter*, *Hafnia-Obesumbacterium*, and *Alistipes*, but also included *Lachnospiraceae*, *Oscillibacter*, *Blautia*, *Akkermansia*, and *Desulfovibrio* (Supplementary Fig. S1). A differential abundance ana­lysis with Aldex revealed that *Oscillibacter* and *Blautia* were differentially abundant genera (considering an effect size >|‍0.8|) between *S. aeneus* and *S. bicanthalis* (effect size of –0.86 and 0.91, respectively), with *Oscillibacter* being more abundant in *S. aeneus* and *Blautia* in *S. bicanthalis* (Fig. 2B). An ordination ana­lysis separated the gut bacterial communities of *S. aeneus* from those of *S. bicanthalis* (Fig. 2C). Accordingly, perMANOVA showed a significant difference in the gut bacterial community structure of *S. aeneus* and *S. bicanthalis* (*F*=1.869, *df*=1, *P*<0.001, adjusted *R*^2^=0.103; Table S3).

### Prediction of metabolic functions

**Fig. 3.**
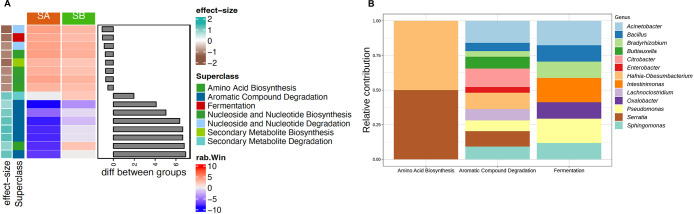
Predicted functions of *Sceloporus aeneus* (SA) and *Sceloporus bicanthalis* (SB). Functional predictions were examined with the ancestral reconstruction algorithm in PICRUSt2. (A) Differentially abundant functions with an effect size >|0.8| as selected by an ANOVA-like differential expression tool for compositional data and Benjamini-Hochberg sequential correction. Median *clr* values (rab.Win) and the effect size were plotted as heatmaps and the median difference of *clr* values between species as bar plots. (B) Bar plots with the relative contribution of bacterial genera to the differential predicted functional pathways.

A total of 1,851 functional genes were predicted and annotated. The most abundant predicted functions across all samples were related to nucleic acid processing. Fifteen functions were different (considering an effect size >|0.8|) between *S. aeneus* and *S. bicanthalis* (Fig. 3A). Functions related to amino acid synthesis and fermentation were more abundant in *S. aeneus* than in *S. bicanthalis*, whereas metabolic functions associated with the degradation of aromatic compounds were more abundant in *S. bicanthalis*. However, none of these predicted functions were significantly different. The ASVs identified as *Hafnia-Obesumbacterium* and *Serratia* equally contributed to amino acid biosynthesis pathways (Fig. 3B). Meanwhile, ASVs belonging to 11 different genera contributed to the degradation of aromatic compounds.

### Comparison of core gut bacterial communities between *Sceloporus* species

**Fig. 4.**
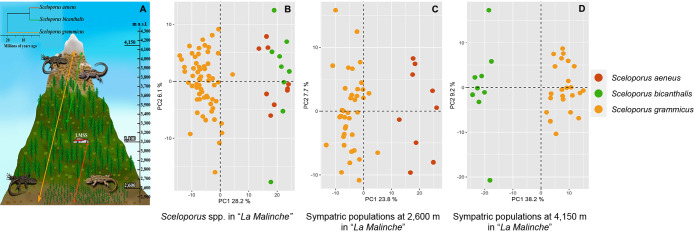
Comparisons of gut bacterial communities of *Sceloporus* species inhabiting a high-mountain ecosystem. (A) Schematic representation of the distribution within La Malinche and phylogenetic relatedness: the lines depict the altitudinal distribution of *Sceloporus grammicus* (orange), *S. aeneus* (red), and *S. bicanthalis* (green). The phylogenetic tree of the three lizard species was represented based on Wiens *et al.* (2013). (B) Principal Component Analysis (PCA) plots using Aitchison dissimilarities of the gut bacterial communities of the three *Sceloporus* lizard species inhabiting the study area, (C) sympatric populations of the *Sceloporus* species at 2,600‍ ‍m a.s.l., and (D) sympatric populations of the *Sceloporus* species at 4,150‍ ‍m a.s.l. in La Malinche. The La Malinche Scientific Station (LMSS) is located at 3,100‍ ‍m a.s.l.

**Fig. 5.**
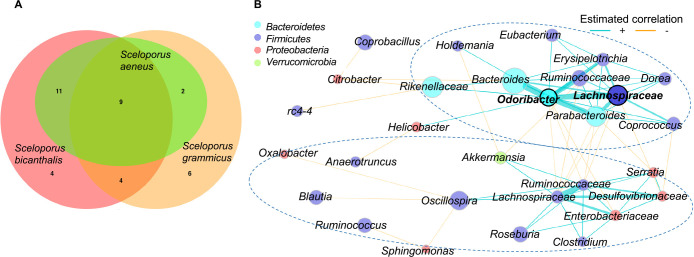
Core gut bacterial communities of *Sceloporus aeneus*, *S. bicanthalis*, and *S. grammicus*. (A) Venn diagram showing the shared and unique core genera of the lizard species and (B) the co-occurrence network of the core-bacterial genera. Nodes represent the bacterial genera and edges show the degree of correlations as obtained with SparCC (≥0.3). Edges in blue are positive correlations and those in orange are negative. Sub-communities, *i.e.*, components, were identified based on a fast greedy modularity optimization algorithm (only components with more than two connected nodes are shown). Nodes with thick black borders were hubs within clusters, *i.e.*, nodes with a connection degree larger than the third quantile within a cluster. Ellipses delimit the components.

Based on the result showing that the gut microbiota of *S. aeneus* was different from that of *S. bicanthalis*, we compared the core gut bacterial biota of these species with that of *S. grammicus*, which coexists with both species at the studied sites (Fig. 4A). The core gut bacterial communities of *Sceloporus* members were separated by species in the ordination ana­lysis (Fig. 4B). Similarly, the population of *S. grammicus* coexisting with *S. aeneus* at 2,600‍ ‍m‍ ‍a.s.l. (Fig. 4C) and *S. grammicus* and *S. bicanthalis* at 4,150‍ ‍m a.s.l. (Fig. 4D) were separated by species in the ordination ana­lysis. The core gut bacterial communities of *S. grammicus* sampled at two different elevations did not significantly differ from each other (*P*>0.05; Table S3), and neither did the core bacterial genera of *S. aeneus* and *S. bicanthalis* (*P*>0.05; Table S3). Core gut bacterial communities were significantly different between *S. grammicus* and *S. aeneus* at 2,600‍ ‍m a.s.l. and between *S. grammicus* and *S. bicanthalis* at 4,150‍ ‍m a.s.l. (*P*<0.05; Table S3). Nine core bacterial genera were shared between the three lizard species, *S. bicanthalis* and *S. aeneus* shared 11 genera, while *S. bicanthalis* had four unique genera and *S. aeneus* had none. *S. grammicus* shared six bacterial genera with both sister species and had six unique genera (Fig. 5A). A co-occurrence network ana­lysis clustered core genera into two components. One of them positively connected *Eubacterium*, *Holdemania*, *Bacteroides*, *Parabacteroides*, *Coprococcus*, and *Dorea* among others, which co-occur in the three lizard species, and the other connected those co-occurring mostly in *S. grammicus* (*Akkermansia*, *Serratia*, *Oscillospira*, *Clostridium*, and *Roseburia* were positively connected and *Ruminococcus*, *Blautia*, and *Sphingomonas* were negatively connected to *Oscillospira*) (Fig. 5B). Both components included members of *Ruminococcaceae* and *Lachnospiraceae*. *Lachnospiraceae* and *Odoribacter* nodes mostly connected the components in the network; therefore, they were identified as hubs. The whole network had a clustering coefficient of 0.53, positive edge percentage of 69.4, and modularity of 0.32.

## Discussion

The present study revealed differences in the composition of the gut microbiota between two closely related lizard species of the genus *Sceloporus* that feed on insects and exhibit similar body sizes and terrestrial habits, but inhabit grasslands with contrasting temperatures and vegetation compositions located at different elevations in La Malinche. *S. bicanthalis*, living in alpine grasslands located at 4,150‍ ‍m‍ ‍a.s.l. with an average temperature of 6.0°C, exhibited greater taxonomic, phylogenetic, and functional alpha diversities in its gut bacterial community than *S. aeneus*, which inhabits cornfields, human-induced grasslands, and shrubs located at 2,600‍ ‍m a.s.l. with an average temperature of 13.2°C. We infer that these differences are mainly driven by non-core bacterial communities and are likely due to differences in food resources.

### Habitats impose different environmental conditions on host species and affect gut bacterial diversity

Specimens of *S. bicanthalis* living at 4,150‍ ‍m a.s.l. must cope with low atmospheric oxygen concentrations, high levels of ultraviolet radiation, and low temperature and humidity levels (Díaz de la Vega-Pérez *et al.*, 2019a; Domínguez-Godoy *et al.*, 2020). These limiting conditions are associated with increased metabolic rates in lizards (Yuni *et al.*, 2015; Plasman *et al.*, 2020), which are required in order to maintain an optimal energetic balance under these conditions (Yuni *et al.*, 2015). Diverse gut microbial communities, particularly short-chain fatty acid-producing bacteria (*e.g.*, *Blautia*, Eubacterium, and *Lachnospiraceae*) that predominate in the gut of *S. bicanthalis*, allow them to satisfy their physiological or energy demands in the challenging environments they occupy at 4,150‍ ‍m a.s.l. (Zhang *et al.*, 2016; Wu *et al.*, 2020). In marked contrast to *S. bicanthalis*, specimens of *S. aeneus* were captured at 2,600‍ ‍m a.s.l., where temperatures are warmer and oxygen availability is higher, and, thus, energy requirements may be lower and diverse gut bacterial communities less important.

Higher diversity in the gut microbiota in *S. bicanthalis* than in *S. aeneus* may also be attributed to parallel differences in diet breadth because high diversity in gut bacterial communities is related to broad diets in reptiles (Hong *et al.*, 2011). However, differences in diet breadth are unlikely to explain the present results. *S. bicanthalis* living at 4,150‍ ‍m‍ ‍a.s.l. may be exposed to a lower diversity of insect prey than *S. aeneus* living at 2,600‍ ‍m a.s.l because insect diversity has been reported to slightly decrease at high elevations elsewhere (McCoy, 1990) and also in La Malinche, as suggested by the number of *Arthropoda* families found in the feces of *S. grammicus* at 4,150‍ ‍m a.s.l. being 10-fold lower than in those living at 2,600‍ ‍m a.s.l. (Montoya-Ciriaco *et al.*, 2020). Nevertheless, diet breadth needs to be estimated for *S. aeneus* and *S. bicanthalis* living at different elevations in order to assess its role as a driver of differences in gut microbiota compositions between these lizard species. The availability of bacterial inoculums acquired from insect prey may be a plausible explanation for the differences observed in gut microbiota compositions between *S. aeneus* and *S. bicanthalis*. Grasslands inhabited by *S. bicanthalis* at 4,150‍ ‍m a.s.l. in La Malinche are less accessible and, thus, less perturbed by human activities (Díaz de la Vega-Pérez *et al.*, 2019b), whereas *S. aeneus* living in cornfields and human-induced grasslands and shrubs at 2,600‍ ‍m a.s.l. is exposed to agrochemicals, including pesticides and chemical fertilizers, which are frequently used to promote growth and protect crops from insects and competitor weeds (García-Juárez *et al.*, 2019). Habitat alterations and exposure to agrochemicals may reduce the diversity of gut bacterial communities in insects (Syromyatnikov *et al.*, 2020), plants (Perazzolli *et al.*, 2014), and animals at higher trophic levels (Amato *et al.*, 2013), and, thus, insects, arachnids, and plant material occasionally eaten by *S. aeneus* (Cruz-Elizalde *et al.*, 2021) may provide less diverse bacterial inoculums than prey eaten by *S. bicanthalis* at less perturbed areas, which, in turn, may translate into differences in the diversity of the gut microbiota.

### Are differences due to different species or habitats?

We compared the core gut microbiota between *S. aeneus* and *S. grammicus* at 2,600‍ ‍m a.s.l. and between *S. bicanthalis* and *S. grammicus* at 4,150‍ ‍m a.s.l. to establish whether differences in the gut bacterial beta diversity between *S. aeneus* and *S. bicanthalis* are due to differences in their environments rather than to differences in species-specific characteristics, such as host genetics, life history, and behavior (Sottas *et al.*, 2021). If environmental conditions are the major driver of the composition of the gut microbiota, no differences in the core gut microbiota were expected between *S. grammicus* and *S. aeneus* coexisting at 2,600‍ ‍m a.s.l. or between *S. grammicus* and *S. bicanthalis* coexisting at 4,150‍ ‍m a.s.l. (we are not aware of areas at which *S. bicanthalis* and *S. aeneus* coexist in La Malinche, and comparisons with *S. grammicus* were the best control we had). In addition, we compared the core microbiota between *S. aeneus* and *S. bicanthalis* to investigate whether more stable gut bacterial communities also differ between these closely related species living in different environments. The core microbiota differed between *S. grammicus* and *S. aeneus* and between *S. grammicus* and *S. bicanthalis*, but not between *S. aeneus* and *S. bicanthalis*. These results are perplexing and imply that dissimilarities in the gut bacterial communities of *S. aeneus* and *S. bicanthalis* are mainly due to differences in non-core bacterial taxa, which are highly influenced by environmental conditions (Grieneisen *et al.*, 2017). Moreover, these results add to evidence for the core microbiota being highly conserved in sister taxa (Baxter *et al.*, 2015; Li *et al.*, 2017), and suggest that differences in overall gut bacterial communities between *S. aeneus* and *S. bicanthalis* may be partially driven by species identity (Baxter *et al.*, 2015; Sottas *et al.*, 2021) because core bacterial communities in the gut differed from those observed in coexisting specimens of *S. grammicus*. Nevertheless, species identity explained only a small part of the variance in gut microbiota compositions between *S. aeneus* and *S. bicanthalis* (R^2^: 0.10), and may account for a small portion of the variance in other iguanian lizards, such as *L. parvus* and *L. ruibali* (R^2^: 0.05) (Kohl *et al.*, 2017). Comparisons of the overall and core gut microbiota between sympatric populations of *S. aeneus* and *S. bicanthalis* will provide insights into the role of ecological factors and species-specific characteristics in the composition of the gut microbiota.

### Differences in taxonomic and functional compositions between lizard hosts

Differences in the composition of the overall gut microbiota between *S. aeneus* and *S. bicanthalis* may be due to genetic differences that these sister species have accumulated since they diverged from their common ancestor ~5.5 million years ago (Wiens *et al.*, 2013). Similarities in their core gut microbiota may be related to a high degree of genetic similarity (Wiens *et al.*, 2010), historically convergent diets (Canseco-Márquez and Gutiérrez-Mayén, 2010), and habitat use (Méndez de la Cruz *et al.*, 2018). The gut bacterial communities of *S. aeneus* and *S. bicanthalis* were dominated by three phyla: *Bacteroidota*, *Firmicutes*, and *Proteobacteria*. These phyla are representative of the bacterial communities of many vertebrates, *e.g.*, birds (Hird *et al.*, 2015) and mammals (Ingala *et al.*, 2018), and, thus, the present results add to evidence for these bacterial phyla maintaining a close and ancient relationship with their vertebrate hosts (Colston and Jackson, 2016). Regarding bacterial genera, the abundance of *Oscillibacter* (*S. aeneus*) and *Blautia* (*S. bicanthalis*) differed between these sister lizards. This pattern is consistent with the higher prevalence of *Blautia* in humans living at high elevations (Han *et al.*, 2021), a bacterial genus associated with short-chain fatty acid production (Liu *et al.*, 2021). Furthermore, *Oscillibacter* was isolated from the gut of the Hawaiian turtle (McDermid *et al.*, 2020) and this genus has been associated with the maintenance of gut barrier integrity (Lam *et al.*, 2012).

Predicted genes involved in the degradation of aromatic compounds were more abundant in *S. bicanthalis* than in *S. aeneus*. Previous studies indicated that under extreme environmental conditions, *Sceloporus* spp. may feed on plant material. Serrano-Cardozo *et al.* (2008) detected plant material in the gastrointestinal tract of *Sceloporus* spp. in a semiarid region of Mexico, while Montoya-Ciriaco *et al.* (2020) identified considerable amounts of the genetic material of plants in the feces of *S. grammicus* in alpine-grasslands. If *S. bicanthalis* feeds on plant material, this may explain the high abundance of functions, such as the degradation of aromatic compounds, but also the large taxonomic, phylogenetic, and functional diversities of the bacterial communities in its digestive tract. In contrast, functions related to amino acid biosynthesis were more frequent in *S. aeneus* than in *S. bicanthalis*. Further research on the metabolism and diet of hosts and the actual functions of bacterial groups is needed to elucidate the underlying causes of this difference; however, one plausible explanation is that bacterial genes associated with carbohydrate and amino acid metabolism may help specimens of *S. aeneus* to process a diet richer in proteins than that of *S. bicanthalis*.

## Conclusion

The present study showed that the taxonomic, phylogenetic, and functional alpha diversities of the gut microbiota were greater in *S. bicanthalis* living at 4,150‍ ‍m a.s.l. than in *S. aeneus* living at 2,600‍ ‍m a.s.l., which may be because more diverse gut bacterial communities allow *Sceloporus* lizards to cope with the limiting conditions that they are exposed to at high elevations (*e.g.*, low temperatures and humidity levels, low atmospheric oxygen concentrations, and high levels of ultraviolet radiation) (Zhang *et al.*, 2016). Differences in the gut microbiota between *S. aeneus* and *S. bicanthalis* appear to mainly be driven by environmentally induced changes in non-core gut bacterial communities; core gut bacterial communities are shared and well conserved in these sister taxa. Further research on the diet and metabolic requirements of *Sceloporus* lizard hosts living at different elevations, and the diversity of bacterial inoculums available in different habitats is warranted to obtain a more detailed understanding of the role of ecological factors as drivers of gut microbiota compositions in wild animals.

### Funding

Funding was provided by Consejo Nacional de Ciencia y Tecnología (CONACyT): Infraestructura project number: 205945, Ciencia de Frontera project number: 137748 and Cátedras CONACyT project number: 883. MH received Ph.D. scholarship number: 967648 and S H-P postdoctoral grant number: 929602 by CONACyT. This article is a requirement for obtaining a Ph.D. degree for the first author.

## Citation

Hernández, M., Ancona, S., Díaz de la Vega-Pérez, A. H.., Muñoz-Arenas, L. C.., Hereira-Pacheco, S. E.., and Navarro-Noya, Y. E.. (2022) Is Habitat More Important than Phylogenetic Relatedness for Elucidating the Gut Bacterial Composition in Sister Lizard Species?. *Microbes Environ ***37**: ME21087.

https://doi.org/10.1264/jsme2.ME21087

## Supplementary Material

Supplementary Material
