# Supplementary material for: Is Habitat More Important than Phylogenetic Relatedness for Elucidating the Gut Bacterial Composition in Sister Lizard Species?
Source: Microbes Environ. 2022 Jun 30;37(3):ME21087. doi: 10.1264/jsme2.ME21087 (PMC9530725; doi:10.1264/jsme2.ME21087)
Supplement: Supplementary file 1 — Supplementary Material [file 37_21087_s1.pdf]

Is Habitat More Important than Phylogenetic Relatedness for Elucidating the Gut Bacterial Composition in Sister Lizard Species?

Mauricio Hernández, Sergio Ancona, Aníbal H. Díaz De La Vega-Pérez, Ligia C. Muñoz-Arenas, Stephanie E. Hereira-Pacheco, and
Yendi E. Navarro-Noya

Supplementary material

Table S1. Summary of the analysis of sequences and dereplication of amplicon sequence variants (ASVs).

Species	Individual ID	No. of raw reads	No. of good quality reads	No. of observed ASVs	Good's coverage (%)[a]
Sceloporus aeneus	Sa.F.D.28	13333	9597	143	99.8
	Sa.F.D.30	11857	9244	147	99.9
	Sa.F.D.33	5611	3829	79	99.5
	Sa.F.D.35	9768	7077	165	99.8
	Sa.M.D.27	4572	2666	104	99.5
	Sa.M.D.29	5643	3654	89	99.6
	Sa.M.D.31	6184	4488	98	99.8
	Sa.M.D.32	7774	5406	143	99.8
	Sa.M.D.36	18409	14503	161	99.7
Sceloporus bicanthalis	Sb.F.D.37	5649	1696	92	98.9
	Sb.F.D.39	11212	8203	169	99.8
	Sb.F.D.44	13446	9575	226	99.8
	Sb.F.D.45	9110	6259	164	99.6
	Sb.M.D.38	9994	7196	166	99.7
	Sb.M.D.41	2165	1300	75	98.8
	Sb.M.D.42	8294	5339	172	99.7
	Sb.M.D.43	7717	5136	144	99.6
	Sb.M.D.46	4519	2751	111	99.3

Good's coverage was calculated with the *QsRutils* R package (Zhang *et al.,* 2017) using the frequency table of ASVs obtained with parameters setting: detect_singletons = FALSE and pool = TRUE to increase sensitivity to rare sequences (Bardenhorst *et al.,* 2022).

Bardenhorst, S.K., Vital, M., Karch, A., Rübsamen, N. (2022) Richness estimation in microbiome data obtained from denoising pipelines. *Comput Struct Biotechnol J* **20**: 508-520.

Zhang, B., Penton, C.R., Xue, C., Quensen, J.F., Roley, S.S., Guo, J. *et al.* (2017) Soil depth and crop determinants of bacterial communities under ten biofuel cropping systems. *Soil Biol Biochem* **112**: 140-152.

Table S2. Mann-Whitney-Wilcoxon test comparison of alpha diversity (Hill numbers) between *Sceloporus aeneus* and *S. bicanthalis*.

Comparison	*W*	*P* value
Taxonomic diversity (*q*=0)	25	0.190
Taxonomic diversity (*q*=1)	9	**0.003**
Taxonomic diversity (*q*=2)	7	**0.001**
Phylogenetic diversity (*q*=0)	24	0.222
Phylogenetic diversity (*q*=1)	9	**0.024**
Phylogenetic diversity (*q*=2)	21	0.093
Functional diversity (*q*=0)	24	0.161
Functional diversity (*q*=1)	7	**0.001**
Functional diversity (*q*=2)	2	**0.000**

Table S3. Permutational analysis of variance (perMANOVA) on Aitchinson distances of the core gut bacterial genera associated with *Sceloporus* species inhabiting a high-mountain ecosystem.

Comparison	*df*	*F*	R^2	*P* value
S. aeneus and *S. bicanthalis* (non-core microbiota)	1	1.84	0.10	**0.001**
S. aeneus and *S. bicanthalis* (core microbiota)	1	1.10	0.06	0.11
All *Sceloporus* species	2	13.91	0.27	**0.001**
S. grammicus and *S. aeneus* at 2600 m a.s.l.	1	11.96	0.21	**0.001**
S. grammicus and *S. bicanthalis* at 4150 m a.s.l.	1	16.98	0.36	0**.001**
Populations of *S. grammicus* at 2600 and 4150 m a.s.l.	1	1.45	0.02	0.13

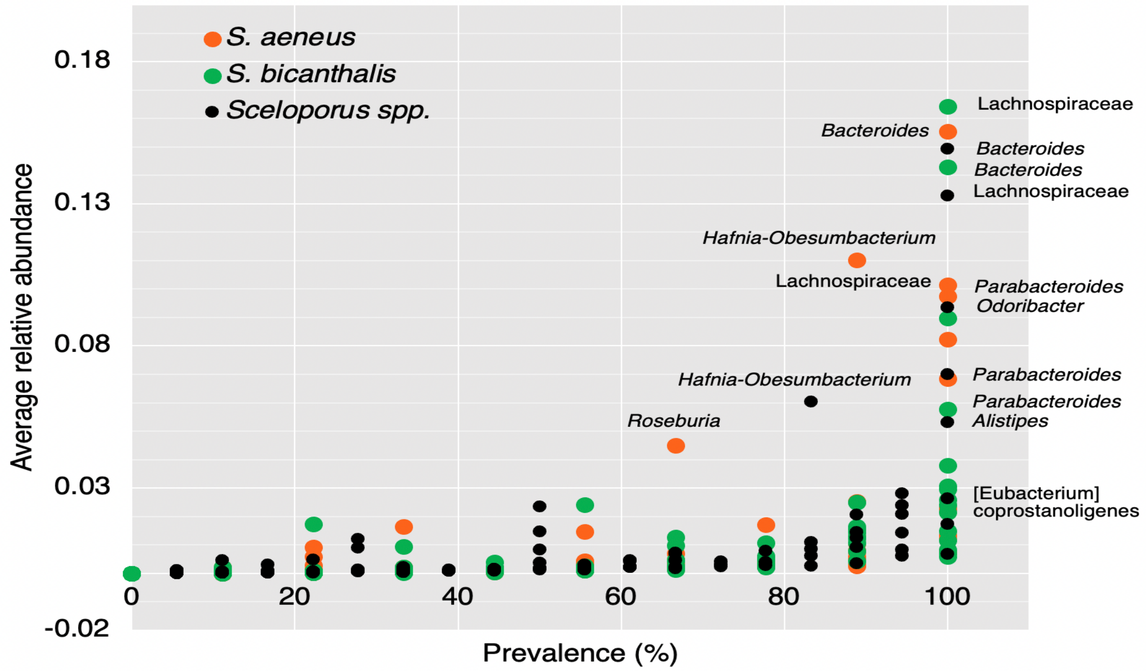

Fig. S1. Relative abundance and prevalence of the gut bacterial genera of three *Sceloporus* lizard species inhabiting a high-mountain ecosystem. The top ten most abundant genera are labeled with their genus names.

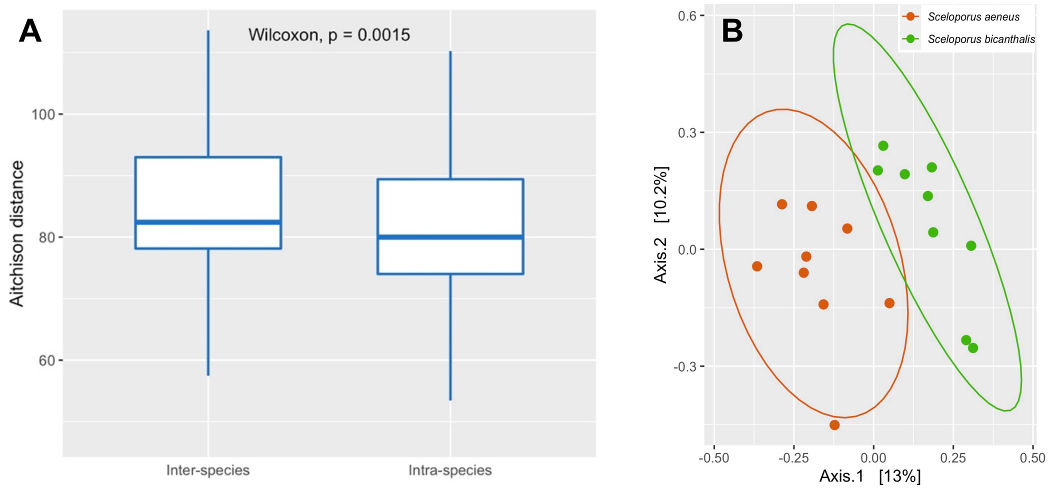

Fig. S2. Dissimilarity based beta diversity analysis of the gut bacterial communities of *Sceloporus aeneus* and *S. bicanthalis*. (A) Box and whisker plots (medians, interquartiles, 10-90% percentiles) of the Aitchison dissimilarities of pairwise comparisons of individuals within the same species (Intra-species) or individual between different species (Inter-species). The significant difference between the two groups was determined with a Mann-Whitney-Wilcoxon test. (B) Principal Coordinate Analysis (PCoA) plots using Bray-Curtis dissimilarities.
